# Extended Follow-up From a Randomized Clinical Trial of Routine Amoxicillin in the Treatment of Uncomplicated Severe Acute Malnutrition in Niger

**DOI:** 10.1001/jamapediatrics.2019.5189

**Published:** 2020-01-13

**Authors:** Sheila Isanaka, Kyra H Grantz, Fatou Berthé, Myrto Schaefer, Eric Adehossi, Rebecca F. Grais

**Affiliations:** 1 Department of Research, Epicentre, Paris, France; 2 Department of Nutrition, Harvard T. H. Chan School of Public Health, Boston, Massachusetts; 3 Department of Global Health and Population, Harvard T. H. Chan School of Public Health, Boston, Massachusetts; 4 Department of Biology and Emerging Pathogens Institute, University of Florida, Gainesville; 5 Epicentre, Niamey, Niger; 6 Médecins Sans Frontières Operational Center Paris, Paris, France; 7 National Hospital, Niamey, Niger

## Abstract

This study is an extended follow-up of a randomized clinical trial of routine amoxicillin use for infants experiencing uncomplicated severe acute malnutrition in Niger.

Evidence to support current guidelines recommending routine antibiotic use in the outpatient management of uncomplicated severe acute malnutrition (SAM) is limited and based largely on data from historical inpatient settings.^1^ The evidence from 2 clinical trials^2,3^ on the effect of routine antibiotic use on nutritional recovery differs. In Malawi, where HIV and kwashiorkor prevalence are high, routine antibiotics increased nutritional recovery and decreased mortality.^2^ In Niger, where HIV and kwashiorkor prevalence are low, we found no benefit of routine amoxicillin on nutritional recovery or mortality, although children receiving amoxicillin had a reduced risk of transfer to inpatient care.^3^

Both reports only considered short-term risks and benefits during nutritional treatment (mean [SD] time to recovery, 29 [19] days in Malawi^2^ and 29 [13] days in Niger^3^), although immunodeficiencies and risk of relapse or morbidity associated with SAM may persist beyond nutritional recovery.^4^ To broaden the available evidence, we present the first analysis (to our knowledge) to assess the outcome of routine antibiotic use in outpatient SAM management, including follow-up during and after nutritional treatment.

## Methods

A complete description of the trial design, procedures, and outcomes has been published previously.^3^ Briefly, children aged 6 to 59 months who presented with SAM in Madarounfa, Niger, between October 2012 and November 2013 were randomly assigned 1:1 to receive amoxicillin (80 mg/kg/d) or a placebo for 7 days. All children received standard care for uncomplicated SAM for a minimum of 3 weeks and a maximum of 8 weeks. Children were followed up weekly for anthropometric, clinical, and vital status during treatment and (as per trial protocol) at 4, 8, and 12 weeks after admission. The Comité Consultatif National d’Ethique, Niger, and the Comité de Protection des Personnes, Île-de-France, France, provided ethical approval. An independent data safety monitoring board reviewed study progress and safety events, and all participants provided written informed consent.

We used the weighted Kaplan-Meier method and Cox proportional hazard models to assess the effect of routine amoxicillin vs placebo on sustained nutritional recovery, transfer to inpatient care, and death from admission to 12 weeks. Inverse probability weights^5^ were used to account for censoring at the time of death or transfer to inpatient care. The intervention outcomes on total anthropometric gains among children who had recovered from admission to 12 weeks were assessed using *t* tests (of weight) and linear regression adjusted for baseline measurements (of mid–upper arm circumference and height). Anthropometric gains over time were estimated by intervention group using hierarchical generalized linear models with a cubic spline.

Data analysis took place from December 2017 to June 2018. Analyses were performed using R, version 3.3 (R Foundation for Statistical Computing). Two-sided *P* values were considered significant at less than .05.

## Results

All 2399 children (mean [SD] age, 16.7 [8.6] months; 1196 female children [49.9%]) of the primary analysis were eligible for inclusion in this extended analysis. Analysis found no association of routine amoxicillin administration with the risk of nutritional recovery, transfer to inpatient care or death, total weight, mid–upper arm circumference, or height gain from admission to 12 weeks (Table). The nutritional and anthropometric benefits of amoxicillin reported previously,^3^ may have been limited to the first 2 to 4 weeks after admission to the nutritional program and not maintained thereafter, including a decreased risk of transfer to inpatient care (cumulative incidence 0-<2 weeks postadmission: amoxicillin group, 8%; placebo group, 9%; 2-12 weeks postadmission: amoxicillin group, 36%; placebo group, 27%) and improved mean (SD) weight gain (during treatment: amoxicillin group, 6.47 [2.65] g/kg/day; placebo group, 5.85 [2.85] g/kg/ day; after program discharge: amoxicillin group, 1.01 [1.12] g/kg/day; placebo group, 1.06 [1.14] g/kg/day) (Figure).

**Table.** Clinical Outcomes and Anthropometric Gains From Admission to 12 Weeks by Intervention Group

Clinical Outcome	No. of Events per Person-Year		Hazard Ratio or Mean Difference (95% CI)	*P* Value
	Amoxicillin	Placebo		
Sustained nutritional recovery	3.26	3.20	0.95 (0.86-1.05)^a^	.36
Transfer to inpatient care	1.72	2.05	0.97 (0.84-1.13)^a^	.70
Death	0.10	0.08	1.11 (0.58-2.13)^a^	.75
Anthropometric gains, mean (SD)				
Weight, g/kg/d	2.82 (1.05)	2.75 (1.02)	0.07 (−0.04 to 0.18)^b^	.21
Mid–upper arm circumference, mm/d	0.18 (0.08)	0.17 (0.07)	0.00 (0.00-0.01)^b^	.22
Height, mm/d	0.19 (0.13)	0.19 (0.13)	0.01 (−0.01 to 0.02)^b^	.41

^a^ Hazard ratios and 95% CIs for amoxicillin relative to placebo are based on the univariate weighted Cox proportional hazard model.

^b^ The mean difference and 95% CIs of anthropometric gains were calculated from unweighted linear regression among children who remained recovered at their final study visit.

**Figure.**
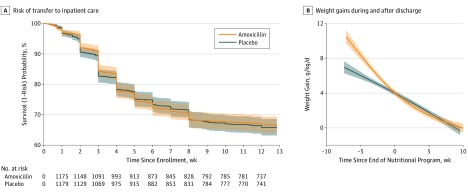
Risk of Transfer to Inpatient Care and Weight Gain During and After Program Discharge A, Weighted Kaplan-Meier estimates of survival (1 − risk) of transfer to inpatient care by intervention group during and after program discharge. Shaded areas are log-transformed 95% CIs. B, Weight gains during and after program discharge by intervention group, estimated using a hierarchical generalized linear model with a cubic spline, including a knot at the time of program discharge. Shaded areas are 95% CIs from 10 000 bootstraps.

## Discussion

Results from this clinical trial with extended follow-up from admission to 12 weeks suggest no longer-term benefit of routine antibiotic use in the treatment of uncomplicated SAM. Current guidelines rightly acknowledge that it would be inappropriate to withhold an intervention that may substantially reduce mortality in a high-risk population,^6^ and in settings where it can save lives, routine antibiotic use should remain part of clinical protocols for uncomplicated SAM. This use, however, should be weighed against the risk of the emergence of antibiotic resistance, implications for program costs and coverage, and likely short-lived individual benefits. Guidance that allows treatment protocols to be adapted and simplified in specific contexts while maintaining individual effectiveness, protecting public health safety, and assuring access to care should be prioritized.
